# Clinical manifestation of norovirus infection in children aged less than five years old admitted with acute diarrhea in Surabaya, Indonesia: a cross-sectional study

**DOI:** 10.12688/f1000research.21069.3

**Published:** 2020-03-09

**Authors:** Alpha Fardah Athiyyah, Katsumi Shigemura, Koichi Kitagawa, Nazara Agustina, Andy Darma, Reza Ranuh, Dadik Raharjo, Toshiro Shirakawa, Masato Fujisawa, Subijanto Marto Sudarmo

**Affiliations:** 1 Department of Child Health, Faculty of Medicine, Airlangga University, Moestopo Street 6-8, Surabaya, 60286, Indonesia; 2 Indonesia-Japan Collaborative Research Center for Emerging and Re-emerging Infectious Diseases, Institute of Tropical Disease, Airlangga University, Mulyorejo Street, Surabaya, 60115, Indonesia; 3 Department of Urology, Kobe University Graduate School of Medicine, 7-5-1 Kusunoki-cho, Chuo-ku, Kobe, 650-0017, Japan; 4 Division of Infectious Diseases, Department of International Health, Kobe University Graduate School of Health Science, 7-10-2 Tomogaoka Suma-ku, Kobe, 654-0142, Japan; 5 Department of Infection Control and Prevention, Kobe University Hospital, 7-5-2 Kusunoki-cho, Chuo-ku, Kobe, 650-0017, Japan; 6 Department of Advanced Medical Science, Kobe University Graduate School of Science, Technology and Innovation, 7-5-1 Kusunoki-cho, Chuo-ku, Kobe, 650-0017, Japan; 7 Institute of Tropical Disease, Airlangga University, Mulyorejo Street, Surabaya, 60115, Indonesia; 8 Center for Infectious Diseases, Kobe University Graduate School of Medicine, 7-5-1, Kusunoki-cho, Chuo-ku, Kobe, 650-0017, Japan

**Keywords:** Diarrhea, Infection, Norovirus, Vesikari score

## Abstract

**Background: **The objective of this study was to investigate the clinical manifestation of norovirus infection between norovirus genogroup and severity of acute diarrhea in pediatric patients at the Dr. Soetomo Hospital, Surabaya, Indonesia.

**Methods: **This cross-sectional study involved 31 participants aged 1-60 months admitted to the hospital with acute diarrhea from April 2012 to March 2013. Norovirus genogroups (GI and II) were identified from patient stool using reverse transcription polymerase chain reaction (RT-PCR). Severity was measured using the Ruuska and Vesikari scoring system.

**Results: **In total, 94 stool samples were obtained, of which 31 (19%) were norovirus positive. Norovirus GI was found in one sample with mild diarrhea. Norovirus GII was found in 30 samples (96.8%); one sample with mild diarrhea (3.3%), 20 samples with moderate diarrhea (66.7%), and nine samples with severe diarrhea (30%).

**Conclusion: **Norovirus GII was the most prevalent cause of acute diarrhea and 30% of the cases manifested as severe diarrhea.

## Introduction

Diarrhea is considered the second-leading cause of death in pediatric patients under the age of five with a worldwide annual mortality of 525,000 children. Diarrhea lasting for even a few days causes dehydration ^1^. Viruses from the genus *Norovirus* from the family *Caliciviridae* are the second-leading cause of acute diarrhea after rotavirus in all age groups of pediatric patients ^2– 4^. Norovirus is responsible for 218,000 pediatric deaths (<15 years old) every year and for 1.1 million pediatric hospitalizations around the world ^2^. In Indonesia, previous studies mentioned incidence rate around 17–30% in children ^5– 8^.

Early identification of norovirus strain and genotype is vital for predicting the development of the disease and selecting the most suitable treatment. Genogroup diversity can be checked using reverse transcriptase polymerase chain reaction (RT-PCR). Norovirus is grouped into 40 viral strains, which are further classified into five different genogroups. Among them, GI and GII possess the most diverse genetic components ^9^. From the previous study, Norovirus GII.2 genotypes had been the most prevalent norovirus strain in Indonesia (71.4%) followed by norovirus GII.17 (14.3%), one case was GII.4 and one case was GII.1 ^10^.

Norovirus commonly causes mild and short-term diarrheal episodes ^10^. Nonetheless, this virus can be fatal, particularly in pediatric, geriatric, and immunocompromised patients ^11, 12^. Norovirus patients showed more severe diarrhea compared to those without norovirus infection in pediatric patients. The type of norovirus strain and genome is thought to be related to diarrhea severity ^12^.

This study aimed to examine the clinical manifestation of norovirus infection in pediatric patients aged 1–60 months in the Dr. Soetomo General Hospital, Surabaya, Indonesia.

## Methods

### Ethical statement

The study protocol was approved by the Ethical Research Commission of Dr. Soetomo General Hospital, Surabaya, and conducted in line with the 1964 Helsinki declaration and its later amendments or research code of ethic issued by the Ministry of Research, Technology and Higher education. Written informed consent regarding participation in this study, the right to resign, stool and data collection and confidentiality of patient data was obtained and signed from all individuals’ parents. Consent was requested from the patients’ parents because the patients were 1–60 months old.

### Study population

This cross-sectional study was conducted between April 2012 and March 2013 of all children aged 1–60 months old with acute diarrhea (described as defecation more than three times per day with change of stool consistency to loose or watery) admitted to the pediatric ward. Patients with a gastrointestinal-anatomical disorder such as Hirschsprung disease, severe systemic disease including sepsis, central nervous system infection or bronchopneumonia, a malabsorption disorder such as cow’s milk allergy, or a compromised immune status were excluded from the study to avoid any bias. On the day of the patients’ admission to the pediatric ward, parents were asked to participate in this study, and they agreed by signing the informed consent form. Stool samples were collected within 24 hours of patient admission with a sterile pot; approximately 3g of stool sample was taken from the middle part of the stool and delivered in no longer than three hours to the laboratory institution. Using a total sampling method, all samples collected until the end of March 2013 were studied.

### Patient assessment

All subjects underwent physical examination and the participant’s parents completed a questionnaire.

The patient assessment was carried out by the physicians. The patient’s parents completed questions in the questionnaire regarding characteristic patient data ^13^. These data were: patient’s identity (age, gender, body weight, and body height); parent’s identity (maternal education); history of diarrhea, which were divided into diarrhea duration (≤4 days, 5 days, and ≥6 days) and diarrhea frequency within 24 hours (1–3 times/day, 4–5 times/day, and ≥6 times/day); vomiting history, divided into vomiting duration (no vomiting, 1 day, 2 days, and more than 3 days) and vomiting frequency within 24 hours (no vomiting, 1 time/day, 2–4 times/day, and more than 5 times/day); and history of breastfeeding (not received breastfeeding, breastfeeding <6 months, and breastfeeding ≥6 months). Nutritional status was classified to either normal or malnutrition (underweight, stunted, wasted, and overweight) according to the definition by WHO ^14^.

All patients also underwent physical examination of axillary body temperature (°C), arterial pulse measured with a pulseoxymeter (times/minute), respiratory rate (times/minute) and inspection of the signs of dehydration based on WHO classification ^15^ and all results were written down in the questionnaire form. The questionnaire was then reviewed by the researchers and entered into the research database.

### Norovirus diagnosis

Stool samples were delivered to the laboratory institution and kept in a deep freezer at -80°C until they were thawed at room temperature prior to RT-PCR analysis. To prevent laboratory contamination, our laboratory staff wore complete apparatuses, such as mask, coat, and gloves, throughout the process. RNA extraction was conducted in Bio Safety Cabinet. Before conducting PCR, all containers were disinfected using alcohol. A 10% stool suspension was prepared for each sample prior to RNA extraction by mixing 100µl stool sample with 100µl phosphate buffered saline buffer (Sigma-Aldrich, St. Louis, USA) with a vortex mixer (QL System, MX-2500 Vortex Mixer, UK) for 15 seconds and then centrifuging at 13,000–15,000 rpm for 10 minutes (Microfuge 20, Beckman Coulter, Indiana Polis, USA). The supernatant (1µl) from the stool suspension was transferred into a clean test-tube and the Viral Nucleic Acid Extraction Kit II (Cat # VR100, Geneaid Biotech Ltd., New Taipei, Taiwan) was used to extract viral RNA, carried out according to the kit manufacturer’s instructions. The eluted RNA from the samples was then stored in a deep freezer at -80°C until RT-PCR processing.

Reverse transcription was performed by mixing 75 picomoles of pdN6 random hexamers (Cat # 11034731001, Roche Molecular Biochemicals, Germany), 4U AMV Reverse Transcriptase (Cat # AMS.AMV007-1, AMS Biotechnology, Abingdon, UK) and 5μl of the eluted RNA and incubating at 42°C for 60 minutes. Approximately 10μl of the previous mixture was added to 5μl distilled water, 3μl Ex Taq DNA Polymerase (RR001B, Takara Bio Inc., Kusatsu, Japan) and 2μl of both forward and reverse primer. The primer pair used in this study for G1 were the G1SKF primer with nucleotide chain CTGCCCGAATTYGTAAATGA targeting nucleotide position 5342-5362 and a product size of 329 bp, and the G1SKR primer, with nucleotide chain CCAACCCARCCATTRTACA targeting nucleotide position 5652-5671 and a product size of 329 bp. The primer pair used in this study for G2 were the G2SKF primer, with nucleotide chain CNTGGGAGGGCGATCGCAA targeting nucleotide position 5058-5077 and a product size of 344 bp, and G2SKR, with nucleotide chain CCRCCNGCATRHCCRTTRTACAT targeting nucleotide position 5378-5401 and a product size of 344 bp.

PCR reaction was performed as follows. Initial denaturation was done at 94°C for 7 minutes, followed by 40 amplification cycles with Takara PCR Thermal Cycler Dice (TP600, Takara Bio Inc.). Each cycle consisted of denaturation at 94°C for 30 seconds, primer annealing at 50°C for 30 seconds for G1 or 57°C for 30 seconds for G2, extension reaction at 72°C for 45 seconds, followed by a final extension for 2 hours 24 minutes. The PCR product was then separated via gel electrophoresis in a 2% agarose gel and visualized under the UV light after ethidium bromide staining. The gel patterns were captured with Printgraph Fx Series (AE-6933FXN, Atto Corporation, Tokyo, Japan). The RT-PCR method used in this study was the one used by Rasanen *et al.* in Finland for identifying norovirus ^16^, which can reveal the genotype variety via nonstructural proteins within the virus ^17^. RT-PCR is considered to have the highest sensitivity for diagnosing norovirus infection compared to other methods ^18^.

### Vesikari Scoring System

The severity of diarrhea was measured using the Vesikari Scoring System (see Table 1). This severity scale was originally developed to evaluate the effectiveness of rotavirus vaccines based on 20 points ^19^. The used parameters have been tested for reliability and validity in a cohort study conducted by Freedman with Cronbach’s α > 0.7.

**Table 1.** Vesikari Scoring System.

Parameter	Score				
	0	1	2	3
**Diarrhea**				
Maximal no. of diarrhea episodes per 24-hour period	0	1–3	4–5	≥6
Diarrhea duration ^17^	0	1–4	5	≥6
**Vomiting**				
Maximal no. of vomiting episodes per 24-hour period	0	1	2–4	≥5
Vomiting duration ^18^	0	1	2	≥3
**Temperature (°C)**	<37.0	37.1–38.4	38.5–38.9	≥39
**Dehydration**	<5%	5–10%	>10%	
**Treatment**	None	Oral rehydration solution	Hospitalization	

Maximum score = 19; score <7 = mild severity; score 7–10 = moderate severity; score >10 = severe severity. This table has been reproduced with reference to the study of Ruuska & Vesikari, 1990 ^19^

Diarrhea severity was assessed by evaluating seven clinical symptoms, including the duration of diarrhea, diarrhea frequency within 24 hours, vomiting duration, vomiting frequency within 24 hours, body temperature, dehydration status, and treatment. From those components, we could use the modified Vesikari score ^20^ to assess diarrhea severity. Mild diarrhea is equal to a score of < 7; a score of 7–10 is equivalent to moderate manifestation, and severe manifestation scores > 10.

### Statistical analysis

Descriptive analysis was used to determine proportions from patients’ and parents’ identity data (age, gender, nutritional status and maternal education variables) and clinical patient data (diarrhea type, diarrhea duration, diarrhea frequency, vomiting duration and frequency, temperature, dehydration status and causative pathogen). The results of basic and clinical data are presented in tables and divided based on the PCR norovirus result (positive norovirus group and negative norovirus group). 

## Results

### Participant characteristics

**Figure 1.**
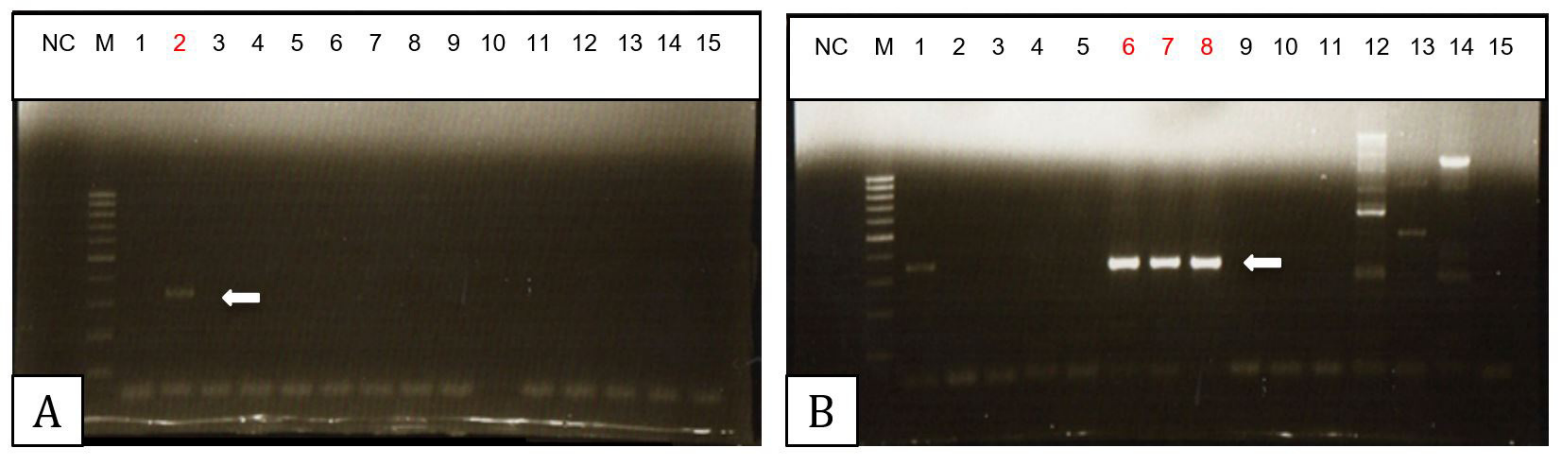
Results of norovirus genogroup analysis by polymerase chain reaction. **A**. Negative control lines (NC), marker lines (M), DNA stepladder marks (100bp). Second lines (GI, 329bp); arrow shows 300 bp marker. **B**. Negative control lines (NC), Marker lines (M), DNA stepladder marks (100bp). Sixth, seventh, and eighth lines (GII, 343bp); arrow shows 300 bp marker.

Samples were collected in the pediatric wards of the Dr. Soetomo General Hospital Surabaya. A total of 94 stool samples were acquired from eligible subjects within 11 months between April 2012 and March 2013. Of those samples, 31 (33.0%) were positive for norovirus infection using the RT-PCR method ( Figure 1) ^13^.

The basic characteristics of all patients participated in this study are presented in Table 2. Most of the participants whose samples were positive for norovirus were male (54.8%), the youngest participant was one month old and the oldest was 24 months old. Twenty-two participants (71%) were between 6–23 months old. As for nutrition status, most of the subjects had adequate nutrition status (67.7%), while 10 subjects (32.3%) were considered malnourished. A total of 26 subjects (83.9%) were breastfed, with 19 subjects (61.3%) breastfed for more than six months and the rest (22.6%) were breastfed for under six months.

**Table 2.** Basic characteristics data.

Variable	Norovirus positive		Norovirus negative	
	n	%	n	%
Age (months)				
1–5	9	29.0	10	15.9
6–23	22	71.0	47	74.6
>23	0	0.0	6	9.5
Gender				
Male	17	54.8	41	65.1
Female	14	45.2	22	34.9
Nutrition status				
Normal	21	67.7	14	22.2
Malnutrition	10	32.3	49	77.8
Breastfeeding status				
Never	5	16.1	3	4.8
Breastfeeding ≤ six months	7	22.6	47	74.6
Breastfeeding ≥ six months	19	61.3	13	20.6
Maternal education				
Low	5	16.1	7	11.1
Middle	22	71.0	47	74.6
High	4	12.9	9	14.3

Most patients in negative norovirus group were within 6–23 months old (74.6%) and were male (65.1%). Differences were found in the nutritional status of norovirus negative patients, in whom malnutrition was more prevalent (77.8%) than in norovirus positive patients. Breastfeeding for less than six months was also more common in the norovirus negative group (74.6%).

Clinical characteristic data are presented in Table 3. On average, the subjects were brought to the hospital after suffering diarrhea for two days with a frequency of diarrhea of five times within 24 hours. Other symptoms experienced by the subjects included vomiting (71% in positive norovirus group and 63% in negative norovirus group) with the most frequent duration of vomiting being one day (14.9% in positive norovirus group and 40.4% in negative norovirus group) and the most commonly observed frequency of vomiting being 2–4 times (9.6% in positive norovirus group and 21.3% in negative norovirus group) per day. The most frequent body temperature on admission to the hospital was below 37°C for positive norovirus group (48.4%) and sub-febrile (37.1–38.4°C) for the negative norovirus group (50.8%). Dehydration status in this study showed that two of the patients suffered from severe dehydration in both groups, while no dehydration was found only in negative norovirus group (3.2%). Watery stool diarrhea was the most frequent type of diarrhea in both positive norovirus group and negative norovirus group (80.6% and 50.4%, respectively). Bloody or mucoid stool was found only in patients of the negative norovirus group (both 9.5%).

**Table 3.** Clinical characteristic data.

Variable	Norovirus positive		Norovirus negative	
	n	%	n	%
Diarrhea type				
Watery	25	80.6	34	54.0
Loose	6	19.4	17	27.0
Bloody	0	0.0	6	9.5
Mucoid	0	0.0	6	9.5
Diarrhea duration				
1–4 days	23	24.5	52	55.3
5 days	3	3.2	7	7.4
≥6 days	5	5.3	4	4.3
Diarrhea frequency				
1–3 times	13	13.8	18	19.1
4–5 times	7	7.4	25	26.6
≥6 times	11	11.7	20	21.3
Experiencing vomiting				
Yes	22	71.0	38	60.3
No	9	29.0	25	39.7
Vomiting duration				
No vomiting	9	9.6	26	26.6
1 days	14	14.9	38	40.4
2 days	2	2.1	0	0
≥3 days	6	6.4	0	0.0
Vomiting frequency				
No vomiting	9	9.6	26	27.7
1 time	7	7.6	10	10.6
2–4 times	9	9.6	20	21.3
≥5 times	6	6.4	7	7.4
Temperature (°C)				
<37.0	15	48.4	27	42.9
37.1–38.4	12	38.7	32	50.8
38.5–38.9	1	3.2	3	4.8
>39	3	9.7	1	1.6
Dehydration status				
No dehydration	0	0.0	2	3.2
Some dehydration	29	93.5	59	93.7
Severe dehydration	2	6.5	2	3.2
Causative pathogen				
Norovirus GI	1	3.2	0	0
Norovirus GII	30	96.8	0	0

### Diarrhea severity

Based on norovirus genogroup identification from gel electrophoresis, GI was found in one sample and GII in 30 samples (96.8%). No products other than norovirus was found.

Distribution between norovirus genogroups and degree of diarrhea severity is presented in Table 4 and shows that norovirus GI was only responsible for one case of diarrhea with moderate severity. From 30 samples that tested positive for norovirus GII, GII was responsible for 20 cases (66.7%) of diarrhea with moderate severity, and nine cases (30%) of diarrhea with severe manifestation.

**Table 4.** Diarrheal severity distribution by norovirus genogroup.

		Norovirus genogroup		Total
		GI	GII	
**Severity**	**Mild (Score <7)**	0 (0%)	1 (3.3%)	1 (3.2%)
	**Moderate (Score 7–10)**	1 (100%)	20 (66.7%)	21 (67.7%)
	**Severe (Score ≥11)**	0 (0%)	9 (30%)	9 (29.1%)
**Total**		1	30	31

## Discussion

Norovirus has been reported as be the main cause of acute diarrhea worldwide after rotavirus in all age groups of pediatric patients both in developed and developing countries ^2, 3^. Norovirus strain type and genome mutation are thought to correlate with the severity of the diarrhea ^11^. It is important to clarify the pathogenesis of this disease to achieve better treatment for each case.

Norovirus was identified in this study in 31 out of 94 samples (33.0%), with norovirus GII in 30 samples (96.8%) and norovirus GI in one sample (3.2%). This agrees with a previous study mentioning norovirus infection was found in 30% of 102 children aged 0–15 months in Jakarta, Indonesia ^5^. However, our study results showed higher norovirus infection incidence than previous studies mentioning incidence of about 17–21% ^6– 8^.

Another study conducted in Rio de Janeiro in Brazil from 2005–2008 showed similar results; 1,087 stool samples obtained from 879 people below 20 years old and 208 people above 20 years old had norovirus in approximately 35% of the samples, and 96% of the norovirus-positive samples were GII positive ^21^. A study of 165 participants in Shanghai, China, also showed high prevalence of norovirus GII infection (97.6%), with only 2.42% of those samples positive for norovirus GI ^22^. These worldwide reports suggest that the most prevalent genogroup infecting humans is GII, with GI only seen in a minority of cases.

Norovirus infection, in our study, is most prevalent in 6–23 months population. Similar to other studies, this finding might be due to protection from maternal antibodies during breastfeeding for infant of < 6 months old. After 2 years of age, incidence of norovirus infection will decline due to acquired immunity ^23– 26^.

The degree of diarrheal severity in subjects infected by norovirus GII was mostly moderate and only 30% were classified as severe. This agrees with a study carried out by Japanese group, Nakagomi *et al.*, confirming that norovirus infection could elicit a similar degree of severity to rotavirus infection ^27^. Similarly, a study in Taiwan showed that norovirus caused mild diarrhea in 30.6%, moderate diarrhea in 43.9% and severe diarrhea in 25.5% of cases using the Vesikari Scoring System. Although previous study found that norovirus GII infection could lead to a more severe clinical manifestation diarrhea and vomiting compared to other genogroups, there are also wide range of severity level within the norovirus GII genogroup itself, such as that norovirus GII.4, GII.2, GII.3, GII.6, and GII.7 are associated with higher severity score ^28^. However, it is still a debate whether the genogroup itself or the viral loads that associate with clinical severity ^29^.

In our study, unfortunately, the degree of diarrheal severity in subjects infected by norovirus GI could not be compared to the degree of diarrheal severity in norovirus GII since norovirus GI was only detected in one sample, which is not enough for comparison.

Although this study achieved its aims, there were unavoidable study limitations. First, our sample size was comparatively small compared to previous norovirus studies in other countries. We did not include neonates below 1 month old due to our limitation to reach the neonatal ward. Secondly, we found no recurrent cases in our study, and therefore we did not analyze the relationship between norovirus genogroup classification and recurrence of diarrhea. Thirdly, since all the patients that participated in this study were all being admitted to the hospital, the treatment criteria are relatively more severe. Nevertheless, our findings largely agree with previous studies in Surabaya, Shanghai, and Rio de Janeiro, as explained in our discussion above ^10, 20, 22^. Fourth, we did not have data about other pathogens, which might be co-infecting. In addition, we could not classify the norovirus GII into genotype, and then use this genotype to infer the severity of the disease. Finally, this study only categorized norovirus genogroups by RT-PCR. We did not perform gene sequencing for norovirus RNA. We also did not include positive controls for each PCR reaction. Future studies will address these limitations.

## Conclusion

This study demonstrated that norovirus was responsible for 33.0% of diarrhea cases in the study group, and norovirus GII was significantly dominant compared to norovirus GI. As many as 30% of norovirus cases had severe diarrheal manifestation, all of which were caused by norovirus GII.

## Data availability

### Underlying data

Harvard Dataverse: Norovirus PCR data set. https://doi.org/10.7910/DVN/KLBUOE ^13^.

This project contains the following underlying data:

- Norovirus PCR data set detaverse.tab (sociodemographic information and clinical findings for norovirus positive patients)
- Master data – Noro negative (1).tab (sociodemographic information and clinical findings for both norovirus negative and positive patients)
- Original PCR gel images in JPEG format

### Extended data

Harvard Dataverse: Norovirus PCR data set. https://doi.org/10.7910/DVN/KLBUOE ^13^.

This project contains the following extended data:

- Norovirus Study Questionnaire (ENG).pdf (copy of questionnaire in English)
- Ind-Informed for Consent_norovirus.pdf (copy of questionnaire in Indonesian)

Data are available under the terms of the Creative Commons Zero “No rights reserved” data waiver (CC0 1.0 Public domain dedication).
