# Supplementary material for: Clinical manifestation of norovirus infection in children aged less than five years old admitted with acute diarrhea in Surabaya, Indonesia: a cross-sectional study
Source: F1000Res. 2020 Mar 9;8:2130. Originally published 2019 Dec 20. [Version 3] doi: 10.12688/f1000research.21069.3 (PMC7065660; doi:10.12688/f1000research.21069.3)
Supplement: Peer review history

# Peer review: Reviewer response for version 3

**DOI:** 10.5256/f1000research.24984.r61038

**Published:** 2020-03-10

**Authors:** Mohamad S. Hakim

**Affiliations:** 1 Department of Microbiology, Faculty of Medicine, Public Health and Nursing, Gadjah Mada University (UGM), Yogyakarta, Indonesia

The authors already addressed all my previous comments.

I confirm that I have read this submission and believe that I have an appropriate level of expertise to confirm that it is of an acceptable scientific standard.

# Peer review: Reviewer response for version 2

**DOI:** 10.5256/f1000research.24539.r60048

**Published:** 2020-02-21

**Authors:** Mohamad S. Hakim

**Affiliations:** 1 Department of Microbiology, Faculty of Medicine, Public Health and Nursing, Gadjah Mada University (UGM), Yogyakarta, Indonesia

The authors already made improvements in this revised version of manuscript based on my previous comments. However, I still have some minor questions:

1. As I have stated earlier in my previous comments, the sample size is too small to perform correlation analysis because only one GI positive manifested as moderate diarrhea. The authors should then not use term “correlation” in the objective (last paragraph of introduction section), as well as in the title. How can the authors make correlation analysis if they had only one GI positive samples? Thus, the term "correlation" is not supported by their data.
  Therefore, I highly recommend to change to a more general title, such as “Clinical manifestation of norovirus infection in children aged less than five years old admitted with acute diarrhea in Surabaya, Indonesia: a cross-sectional study” or “Norovirus infection in children aged less than five years old admitted with acute diarrhea in Surabaya, Indonesia: a cross-sectional study”.
2. The authors mentioned, “Genogroup diversity can be checked using immunochromatography and reverse transcriptase polymerase chain reaction (RT-PCR)". Previously I was only concerned about electron microscopy, but I just realized that is it true that genogroup diversity can also be determined by immunochromatography? Please support your statement with solid references.
3. Please change “severer” to “more severe” (in introduction section). I have mentioned this previously, but the authors did not make any changes. 
4. In the Discussion, the authors mentioned, “We did not perform gene sequencing for norovirus DNA.”
  Comment: Norovirus is an RNA virus, not DNA virus. Please change. 
5. In the Discussion, the authors mentioned, “One of the possible contributing factors is because we did not use positive controls for our PCR; therefore our result might have high false positive result.”
  Comment: high rate of false positive result mostly due to unproper **negative **control, not positive control or due to high contaminations. In contrast, positive control is employed to reduce false negative results. The best way to confirm the PCR results is by performing sequencing of some or all positive samples. The authors should modify this issue in the next revision.

I confirm that I have read this submission and believe that I have an appropriate level of expertise to confirm that it is of an acceptable scientific standard, however I have significant reservations, as outlined above.

# Peer review: Reviewer response for version 2

**DOI:** 10.5256/f1000research.24539.r60047

**Published:** 2020-02-17

**Authors:** Hirokazu Kimura

**Affiliations:** 1 Department of Health Science, Graduate School of Health Science, Gunma Paz University, Gunma, Japan

The authors addressed all my comments. Thus, I recommend that the revised manuscript is now suitable for indexing.

I confirm that I have read this submission and believe that I have an appropriate level of expertise to confirm that it is of an acceptable scientific standard.

# Peer review: Reviewer response for version 1

**DOI:** 10.5256/f1000research.23188.r57998

**Published:** 2020-01-27

**Authors:** Hirokazu Kimura

**Affiliations:** 1 Department of Health Science, Graduate School of Health Science, Gunma Paz University, Gunma, Japan

The authors studied the correlation between NoV genogroup and severity of acute diarrhea in the pediatric patients with gastroenteritis at the Hospital, Surabaya, Indonesia. NoV was detected in around 20% of the patients. In many cases, GII virus was detected, while GI was detected in one case. Moreover, 30% of the patients showed severe diarrhea. Overall, the manuscript was well written, while I had some minor comments.

1. The authors only examined by RT-PCR method. Did you detect nonspecific PCR products in the amplicons? Please add it in the results.
2. How did you prevent laboratory contamination? Please provide it in the revised manuscript.            

I confirm that I have read this submission and believe that I have an appropriate level of expertise to confirm that it is of an acceptable scientific standard, however I have significant reservations, as outlined above.

# Response

**Published:** 2020-01-29

**Authors:** Katsumi Shigemura

**Affiliations:** Kobe University Hospital, Japan

**1.** **The authors only examined by RT-PCR method. Did you detect non-specific PCR products in the amplicons? Please add it in the results.**

Response: We suggest that non-specific PCR products refer to viruses other than norovirus. If that so, there were no products other than norovirus was found.

**2.** **How did you prevent laboratory contamination? Please provide it in the revised manuscript.**

Response: To prevent laboratory contamination, our laboratory staff wore complete apparatuses, such as mask, coat, and gloves. RNA extraction was conducted in Bio Safety Cabinet. Before conducting PCR, all containers were disinfected using alcohol.

# Peer review: Reviewer response for version 1

**DOI:** 10.5256/f1000research.23188.r57997

**Published:** 2020-01-06

**Authors:** Mohamad S. Hakim

**Affiliations:** 1 Department of Microbiology, Faculty of Medicine, Public Health and Nursing, Gadjah Mada University (UGM), Yogyakarta, Indonesia

**Summary:**

 Athiyyah *et al*. reported the prevalence of norovirus infection during one year study of acute diarrhea patients <5 years old admitted to Dr. Soetomo Hospital, Surabaya, Indonesia. Reports of norovirus prevalence in children <5 years old with acute diarrhea in Indonesia are still limited. Therefore, this paper is highly important to expand the data of norovirus surveillance in Indonesia.

**Comments:**

 However, this paper needs a major revision to improve the way of presenting and discussing the data as I recommend the following:

1. The sample size is too small to perform correlation analysis. Only one GI positively manifested as moderate diarrhea. The authors should then not use “correlation” in the objective (last paragraph of introduction section), as well as in the title, this does not make any sense. The author can change into a more general title, such as “Clinical manifestation of norovirus infection in children aged less than five years old admitted with acute diarrhea in Surabaya, Indonesia: a cross-sectional study” or “Norovirus infection in children aged less than five years old admitted with acute diarrhea in Surabaya, Indonesia: a cross-sectional study.”
2. The authors mention in the introduction: “Genogroup diversity can be checked using several methods including immunochromatography, reverse transcriptase polymerase chain reaction (RT-PCR), and electron microscopy.”
  Comments: any supporting evidence or reference of this statement? Norovirus genogroup is based on genetic analysis, so somehow it is weird that it can be differentiated based on electron microscopy.
3. The authors did not perform comprehensive literature searching of the previous norovirus study in Indonesia. In fact, there are already some “key” publications in this field, but the authors did not include them in this manuscript.
  Please thoroughly check the referenced papers to improve author statement in both introduction and discussion section ^1^^-^^5^.
4. Please change “severer” to “more severe” (in introduction section).
5. In the methods: Are there any specific reason to exclude <1 month baby in this study?
6. Figure 2B, lane 1. Is it considered positive or negative? Because it seems a scanty, positive band there. It looks like Figure 2A, lane 2 which is considered as positive for norovirus GI.
7. Do the authors include positive controls for each PCR reaction? The prevalence of norovirus in this study (about 32%) is higher than that of global prevalence (about 20%) in countries that did not include Rotavirus vaccination in the NIP. So the authors should ensure that a proper and valid PCR assay has been conducted. Please check: Ahmed SM *et al*. (2014) ^6^.
8. Figure 1 is not necessary, so please delete it.
9. The authors mention: “Of those samples, 31 were positive for norovirus infection using the RT-PCR method”. Please provide the percentage of norovirus-positive samples.
10. The authors mention in discussion: “This agrees with a previous study done in healthy subjects in Surabaya, Indonesia…" This is not a match comparison, because the previous study is in healthy, asymptomatic adult subjects. The authors should check the above papers that I recommended for much better comparison of previous studies in Indonesia.
11. In the discussion, the authors should also discuss associated risk factors of contracting norovirus based on Table 2. For example, why did in your population is the most prevalent age of norovirus positive 6-23 months?
12. The authors should add discussion about different severity of norovirus infection based on different genotpe of GII norovirus. Although they did not perform genotype identification in this study, the readers of this paper should still be aware that different GII genotypes can cause different severity of clinical manifestations.
13. In conclusion, it is mentioned: “This study demonstrated that norovirus was responsible for 48.4% of diarrhea cases in the study group”. Could the authors clarify about the percentage of 48.4%? 31 out of 94 should be 32.9%. It is also not consistent with the abstract (I mention below).
14. Reference no. 6, what journal that publishes this paper? The authors did not mention this. If this is not published in a peer-reviewed journal, it would be better to change the reference with one I recommended.
15. The abstract: “This cross-sectional study involved 31 participants” --> please delete “31”. When you design the study, you never know how many patients will participate.
  “In total, 91 stool samples were obtained, of which 31 (19%) … “ --> please clarify the percentage. Also, is it 91 or 94? Please thoroughly check all the numbers and percentages you mentioned in this paper.
16. Table 2, change “Malutrition” to “Malnutrition”. Also, please describe in the methods what the criteria to categorize this nutrition status are.

I confirm that I have read this submission and believe that I have an appropriate level of expertise to state that I do not consider it to be of an acceptable scientific standard, for reasons outlined above.

## References

[1] : Norovirus and rotavirus infections in children less than five years of age hospitalized with acute gastroenteritis in Indonesia. *Arch Virol*.2019;164(6) : 10.1007/s00705-019-04215-y 1515-1525 10.1007/s00705-019-04215-y 30887229

[2] : Enteropathogens associated with acute diarrhea in community and hospital patients in Jakarta, Indonesia. *FEMS Immunol Med Microbiol*.2002;34(2) : 10.1111/j.1574-695X.2002.tb00615.x 139-46 10.1111/j.1574-695X.2002.tb00615.x 12381465

[3] : Incidence of Norwalk-like viruses, rotavirus and adenovirus infection in patients with acute gastroenteritis in Jakarta, Indonesia. *FEMS Immunol Med Microbiol*.2002;33(1) : 10.1111/j.1574-695X.2002.tb00568.x 27-33 10.1111/j.1574-695X.2002.tb00568.x 11985965

[4] : Genotyping and clinical factors in pediatric diarrhea caused by rotaviruses: one-year surveillance in Surabaya, Indonesia. *Gut Pathog*.2015;7: 10.1186/s13099-015-0048-2 3 10.1186/s13099-015-0048-2 25793014PMC4365806

[5] : Significance of continuous rotavirus and norovirus surveillance in Indonesia. *World J Pediatr*.14(1) : 10.1007/s12519-018-0122-1 4-12 10.1007/s12519-018-0122-1 29446040

[6] : Global prevalence of norovirus in cases of gastroenteritis: a systematic review and meta-analysis. *Lancet Infect Dis*.2014;14(8) : 10.1016/S1473-3099(14)70767-4 725-730 10.1016/S1473-3099(14)70767-4 24981041PMC8006533

# Response

**Published:** 2020-01-31

**Authors:** Katsumi Shigemura

**Affiliations:** Kobe University Hospital, Japan

**RESPONSES**

We greatly appreciate your complimentary comments and suggestions.

1. We apologize that our sample size is too small, however, from this limited sample still we could explore many things.

2. We apologize that electron microscopy is not a method could be used for genotyping. We will revise the manuscript to: Genogroup diversity can be checked using immunochromatography and reverse transcriptase polymerase chain reaction (RT-PCR).

3. We will check the suggested reference and revise our manuscript accordingly.

4. We will revise the manuscript accordingly.

5. We exclude neonates below 1 month old because of our limitation to involve neonates admitted in the neonatal wing to follow the study.

6. Figure 2B lane 1 is considered positive.

7. It is our limitation that we do not include positive controls for each PCR reaction.

8. We will revise the manuscript accordingly.

9. We involve 94 subjects in this study and 31 subjects (32.9%) were norovirus positive.

10. We will check the suggested reference and revise our manuscript accordingly. Our study result showed higher norovirus infection incidence than previous studies mentioning incidence about 17-21% (Oyofo *et al.*, 2002; Subekti *et al.*, 2002; Nirwati *et al.*, 2019). One of the possible contributing factors is because we do not use positive controls for our PCR.

11. We will revise our manuscript accordingly. Norovirus infection is most prevalent in 6-23 months due to protection from maternal antibodies during breastfeeding for infant < 6 months old. After 2 years of age, cases of norovirus will decline due to acquired immunity (Japhet *et al.*, 2012; Trang *et al.*, 2012; El Qazoui *et al.*, 2014; Mikounou Louya *et al.*, 2019).

12. Previous studies have shown different severity in the clinical manifestation of Norovirus GII. Genogroup of Norovirus GII.4, GII.2, GII.3, GII.6, and GII.7 are associated with higher severity score (Mathew *et al.*, 2019). However, it is also difficult to determine whether genogroups or viral loads that associated with clinical severity (Chan *et al.*, 2015)

13 We involve 94 subjects in this study and 31 subjects (32.9%) were norovirus positive. We will revise our manuscript.

14. We will check the suggested reference and revise our manuscript accordingly.

15. We will revise our manuscript accordingly.

16. We will revise our manuscript accordingly. Nutrition status is based on WHO curve for children aged 1-60 months.

We will check the suggested reference and revise our manuscript accordingly. Thank you very much for reviewing our manuscript.

**Reference**

Chan, M. C. W. *et al.* (2015) ‘Virus Genotype Distribution and Virus Burden in Children and Adults Hospitalized for Norovirus Gastroenteritis, 2012-2014, Hong Kong.’, *Scientific reports*, 5, p. 11507. doi: 10.1038/srep11507.

Japhet, M. O. *et al.* (2012) ‘Molecular epidemiology of rotavirus and norovirus in Ile-Ife, Nigeria: High prevalence of G12P[8] rotavirus strains and detection of a rare norovirus genotype’, *Journal of Medical Virology*, 84(9), pp. 1489–1496. doi: 10.1002/jmv.23343.

Mathew, S. *et al.* (2019) ‘Epidemiological, Molecular, and Clinical Features of Norovirus Infections among Pediatric Patients in Qatar’, *Viruses*, 11(5), p. 400. doi: 10.3390/v11050400.

Mikounou Louya, V. *et al.* (2019) ‘Molecular characterization of norovirus infection responsible for acute diarrhea in Congolese hospitalized children under five years old in Brazzaville, Republic of Congo’, *International Journal of Infectious Diseases*, 88, pp. 41–48. doi: 10.1016/j.ijid.2019.07.034.

Nirwati, H. *et al.* (2019) ‘Norovirus and rotavirus infections in children less than five years of age hospitalized with acute gastroenteritis in Indonesia’, *Archives of Virology*, 164(6), pp. 1515–1525. doi: 10.1007/s00705-019-04215-y.

Oyofo, B. A. *et al.* (2002) ‘Enteropathogens associated with acute diarrhea in community and hospital patients in Jakarta, Indonesia’, *FEMS Immunology & Medical Microbiology*, 34(2), pp. 139–146. doi: 10.1111/j.1574-695X.2002.tb00615.x.

El Qazoui, M. *et al.* (2014) ‘Rotavirus and Norovirus infections among acute gastroenteritis children in Morocco’, *BMC Infectious Diseases*, 14(1), p. 300. doi: 10.1186/1471-2334-14-300.

Subekti, D. *et al.* (2002) ‘Incidence of Norwalk-like viruses, rotavirus and adenovirus infection in patients with acute gastroenteritis in Jakarta, Indonesia.’, *FEMS immunology and medical microbiology*, 33(1), pp. 27–33. doi: 10.1111/j.1574-695X.2002.tb00568.x.

Trang, N. V. *et al.* (2012) ‘Detection and molecular characterization of noroviruses and sapoviruses in children admitted to hospital with acute gastroenteritis in Vietnam’, *Journal of Medical Virology*, 84(2), pp. 290–297. doi: 10.1002/jmv.23185.

Chan, M. C. W. *et al.* (2015) ‘Virus Genotype Distribution and Virus Burden in Children and Adults Hospitalized for Norovirus Gastroenteritis, 2012-2014, Hong Kong.’, *Scientific reports*, 5, p. 11507. doi: 10.1038/srep11507.

Japhet, M. O. *et al.* (2012) ‘Molecular epidemiology of rotavirus and norovirus in Ile-Ife, Nigeria: High prevalence of G12P[8] rotavirus strains and detection of a rare norovirus genotype’, *Journal of Medical Virology*, 84(9), pp. 1489–1496. doi: 10.1002/jmv.23343.

Mathew, S. *et al.* (2019) ‘Epidemiological, Molecular, and Clinical Features of Norovirus Infections among Pediatric Patients in Qatar’, *Viruses*, 11(5), p. 400. doi: 10.3390/v11050400.

Mikounou Louya, V. *et al.* (2019) ‘Molecular characterization of norovirus infection responsible for acute diarrhea in Congolese hospitalized children under five years old in Brazzaville, Republic of Congo’, *International Journal of Infectious Diseases*, 88, pp. 41–48. doi: 10.1016/j.ijid.2019.07.034.

Nirwati, H. *et al.* (2019) ‘Norovirus and rotavirus infections in children less than five years of age hospitalized with acute gastroenteritis in Indonesia’, *Archives of Virology*, 164(6), pp. 1515–1525. doi: 10.1007/s00705-019-04215-y.

Oyofo, B. A. *et al.* (2002) ‘Enteropathogens associated with acute diarrhea in community and hospital patients in Jakarta, Indonesia’, *FEMS Immunology & Medical Microbiology*, 34(2), pp. 139–146. doi: 10.1111/j.1574-695X.2002.tb00615.x.

El Qazoui, M. *et al.* (2014) ‘Rotavirus and Norovirus infections among acute gastroenteritis children in Morocco’, *BMC Infectious Diseases*, 14(1), p. 300. doi: 10.1186/1471-2334-14-300.

Subekti, D. *et al.* (2002) ‘Incidence of Norwalk-like viruses, rotavirus and adenovirus infection in patients with acute gastroenteritis in Jakarta, Indonesia.’, *FEMS immunology and medical microbiology*, 33(1), pp. 27–33. doi: 10.1111/j.1574-695X.2002.tb00568.x.

Trang, N. V. *et al.* (2012) ‘Detection and molecular characterization of noroviruses and sapoviruses in children admitted to hospital with acute gastroenteritis in Vietnam’, *Journal of Medical Virology*, 84(2), pp. 290–297. doi: 10.1002/jmv.23185.
